# Recovery from fulminant immune-related myocarditis induced by ipilimumab plus nivolumab in malignant pleural mesothelioma: a case report

**DOI:** 10.1186/s12890-026-04193-3

**Published:** 2026-03-10

**Authors:** Yuki Konya, Junko Tanizaki, Kyohei Onishi, Hiroki Matsuzoe, Hiroaki Kanemura, Takayuki Takahama, Kaoru Tanaka, Gaku Nakazawa, Hidetoshi Hayashi

**Affiliations:** 1 Department of Medical Oncology, Kindai University Faculty of Medicine, 1-14-1 Mihara-dai, Minami-ku, Sakai, Osaka, 590-0197 Japan; 2 Department of Cardiovascular Medicine, Kindai University Faculty of Medicine, 1-14-1 Mihara-dai, Minami-ku, Sakai, Osaka, 590-0197 Japan

**Keywords:** Immune checkpoint inhibitors, Ipilimumab, Nivolumab, Immune-related adverse events myocarditis, Malignant pleural mesothelioma

## Abstract

**Background:**

Immune checkpoint inhibitors (ICIs) have become standard therapies for various cancers; however, their use can lead to diverse immune-related adverse events (irAEs). While most irAEs are mild, some, such as myocarditis, although rare, have a high mortality rate. This report highlights a case in which aggressive and invasive therapeutic interventions led to successful patient survival after fulminant ICI-induced myocarditis.

**Case presentation:**

A 64-year-old male with a malignant pleural mesothelioma was admitted to the hospital with dyspnea on day 22 after the first cycle of ipilimumab plus nivolumab. Upon admission, he presented with cardiogenic shock and pulseless ventricular tachycardia, accompanied by elevated cardiac biomarkers. Owing to a history of ICI treatment, immune-related myocarditis was suspected, and high-dose methylprednisolone was immediately initiated. As his hemodynamic status rapidly deteriorated, extracorporeal membrane oxygenation (VA-ECMO), percutaneous left ventricular assist device (pLVAD), and temporary transvenous cardiac pacing were promptly initiated to stabilize the circulation. A myocardial biopsy confirmed lymphocytic myocarditis. The patient gradually stabilized and was successfully weaned off aggressive circulatory support, leading to patient’s discharge on day 69.

**Conclusions:**

Early methylprednisolone pulse therapy is crucial in treating immune-related myocarditis. To bridge the time until the steroids become fully effective, aggressive interventions such as VA-ECMO, pLVAD, and external pacing can play a life-saving supportive role. This underscores the importance of close collaboration with cardiologists to promptly implement these bridging therapies, which are vital for patient survival.

**Supplementary Information:**

The online version contains supplementary material available at 10.1186/s12890-026-04193-3.

## Background

Immune checkpoint molecules, such as PD-1, PD-L1, and CTLA-4, are expressed on immune and cancer cell surfaces, where they function to transmit inhibitory signals to the immune system. Immune checkpoint inhibitors (ICIs) bind to these molecules and block inhibitory signal transmission, thereby sustaining T cell activation and enabling them to attack cancer cells. Ipilimumab, an anti-CTLA-4 antibody, in combination with nivolumab, an anti-PD-1 antibody, is used as first-line treatment for malignant pleural mesothelioma and has been shown to significantly improve survival [1]. Although ICIs have become a standard treatment option, they can induce a wide range of immune-related adverse events (irAEs). Although most irAEs are mild, there have been reports of severe and potentially fulminant irAEs. One such severe irAE is myocarditis; even though the incidence of immune-related myocarditis is only 0.06–1.14% [2], it has a high mortality rate of up to 50% [3]. Here, we report a case of ICI-induced myocarditis that was successfully managed with aggressive and invasive treatment, resulting in patient survival.

## Case presentation

A 64-year-old male with a smoking history of 20 cigarettes per day for 20 years and asbestos exposure was diagnosed with epithelial-type malignant pleural mesothelioma. (StageIV cT4N0M1a) In May 2024, nivolumab (360 mg every 3 weeks) plus ipilimumab (1 mg/kg every 6 weeks) combination therapy was initiated. On day 22 of the first cycle, the patient developed dyspnea at 5 a.m. and, upon no improvement in symptoms, was transported to the emergency department of our institution at 7 a.m.

**Fig. 1:**
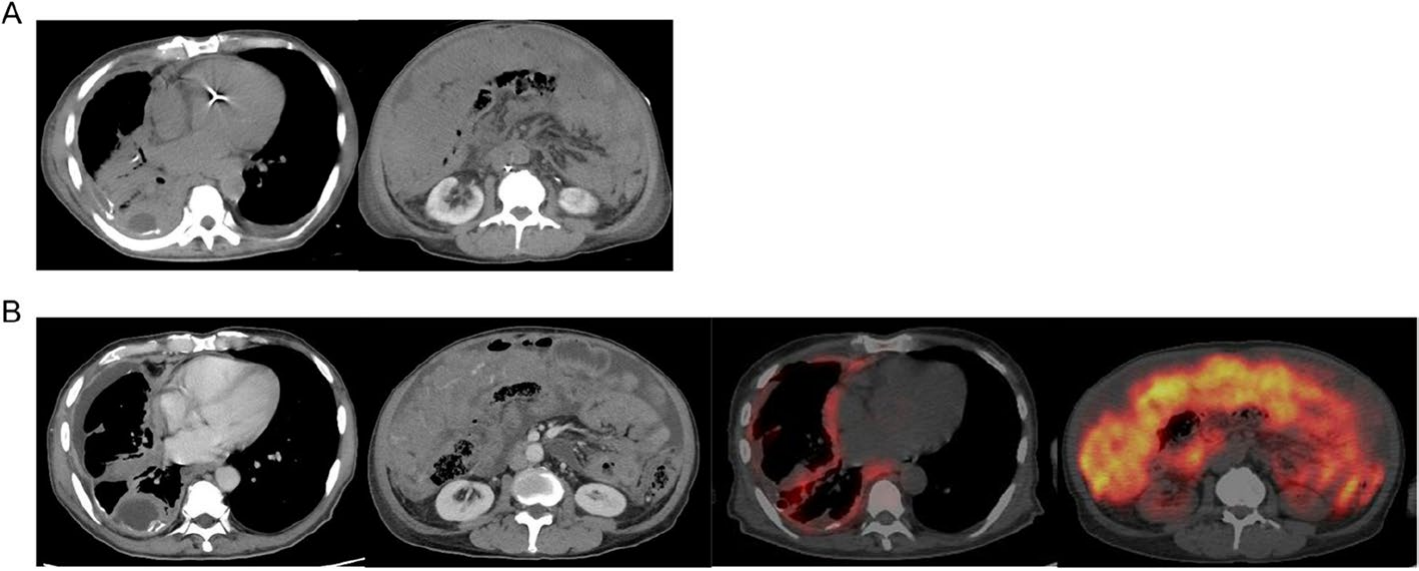
Baseline and post-treatment imaging. **a** CT (computed tomography) showing right pleural thickening, peritoneal dissemination, tumor-like soft tissue shadows, and ascites at the time of presentation to the emergency department. **b** Baseline CT and PET showing right pleural thickening, peritoneal dissemination, tumor-like soft tissue shadows, and ascites at baseline

**Fig. 2:**
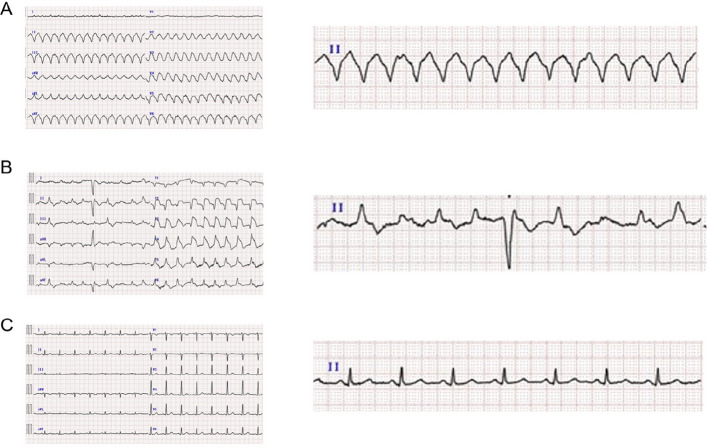
Electrocardiography (ECG) findings. **a** Initial ECG in the emergency department demonstrating pulseless ventricular tachycardia. **b** ECG after direct current-cardioversion, showing persistent ST elevation. **c** ECG obtained before initiation of immune checkpoint inhibitor (ICI) treatment, showing normal sinus rhythm without conduction abnormalities

Upon admission, the patient presented with the following findings: body temperature of 35.4 °C, low blood pressure (unmeasurable), heart rate of 170 bpm, respiratory rate of 26 breaths/min, and SpO_2_ of 70% on room air. Blood tests revealed mild elevation of inflammatory markers, such as C-reactive protein, and white blood cell count. The levels of cardiac biomarkers such as creatine kinase-MB (CK-MB) and troponin I were also elevated. Computed tomography (CT), electrocardiography (ECG), and echocardiography were performed as diagnostic workups. CT showed right pleural thickening, peritoneal dissemination, tumor-like soft tissue shadows, and ascites (Fig. 1a), with no obvious disease progression compared to the baseline scans before treatment initiation (Fig. 1b). ECG revealed pulseless ventricular tachycardia (Fig. 2a), and synchronized direct-current cardioversion was performed. However, the ST elevation persisted (Fig. 2b). The patient did not show any abnormal electrocardiographic findings prior to treatment (Fig. 2c). On echocardiography, the left ventricular ejection fraction was 60%, indicating preserved systolic function. However, the left ventricle appeared compressed by the interventricular septum, resulting in a D-shaped appearance. To rule out ischemic heart disease and, more importantly, immune-related myocarditis, which warranted primary consideration given the patient’s treatment history, coronary angiography and endomyocardial biopsy were performed. However, no significant stenosis was observed in either coronary artery. In addition, although the presence of a D-shaped left ventricle raised concern for right ventricular pressure overload, computed tomography revealed no findings suggestive of pulmonary embolism, and there was no marked elevation in D-dimer levels. Therefore, the likelihood of acute pulmonary embolism was considered to be low. Following a discussion between cardiologists and medical oncologists, high-dose methylprednisolone (mPSL) therapy (1000 mg for three days on the same day without waiting for biopsy results) was administered. Note that the initial dyspnea had appeared at 5 a.m., and the treatment intervention was conducted at 11 a.m. on the same day, allowing for treatment intervention within 6 h of symptom onset.

**Fig. 3:**
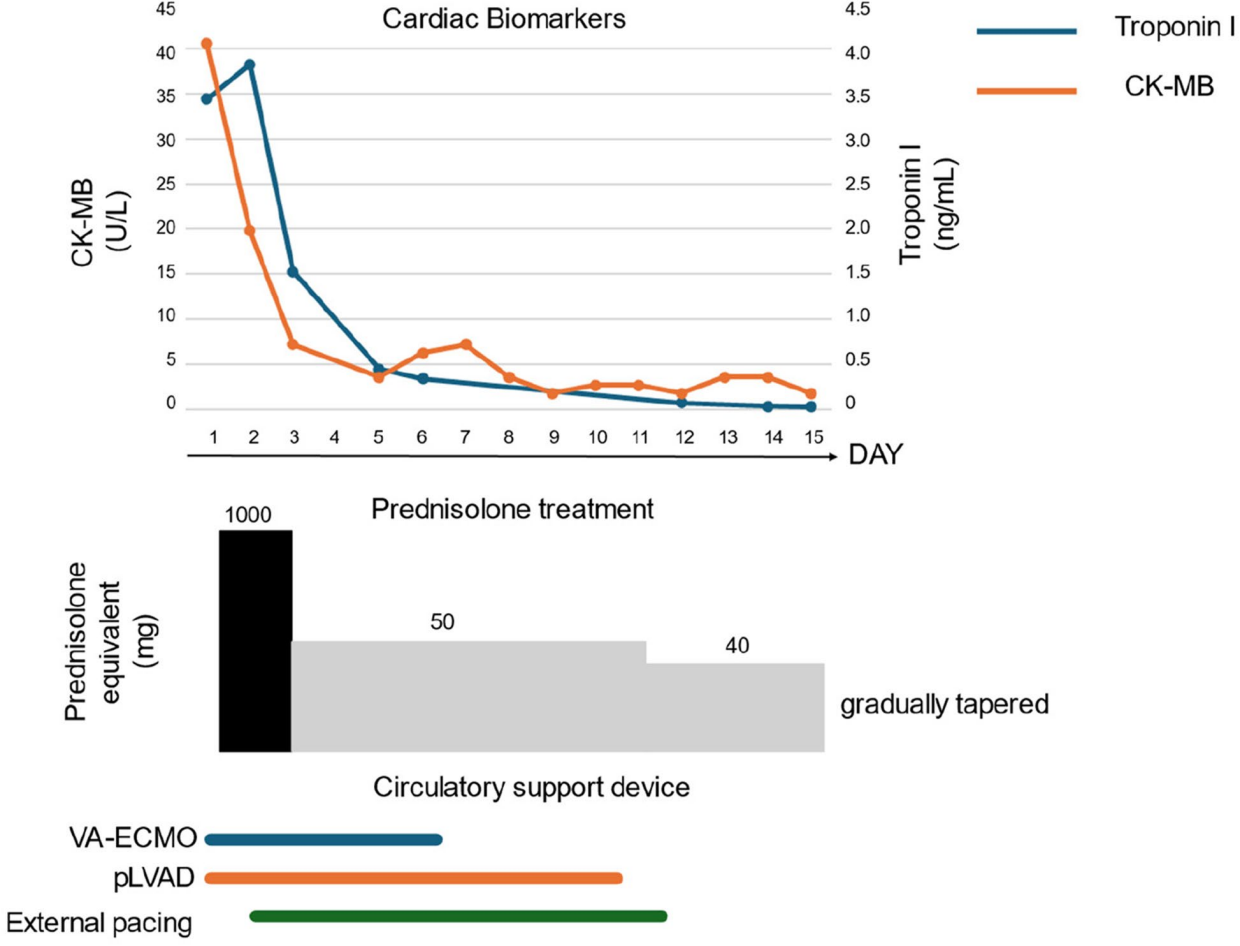
Clinical course, biomarker trends, and mechanical circulatory support. Timeline depicting corticosteroid dosing, invasive mechanical circulatory support, and serial serum Troponin I and creatine kinase-MB (CK-MB) concentrations

Within a few hours of mPSL initiation, the patient’s circulatory status worsened and recurrent ventricular arrhythmia developed. In addition to the right-sided heart failure, the left ventricular ejection fraction decreased to 22%, resulting in biventricular failure. Consequently, advanced mechanical circulatory support was initiated, including extracorporeal membrane oxygenation (VA-ECMO), a percutaneous left ventricular assist device (pLVAD), Impella CP® (Abiomed, Danvers, MA), all under endotracheal intubation. The external pacing was initiated in response to the sudden onset of atrioventricular block to prevent bradycardia, with the pacing system configured to allow overdrive pacing for the prevention of recurrent ventricular arrhythmias. Regarding pharmacological therapy, norepinephrine and dobutamine were administered as vasopressors prior to the initiation of VA-ECMO, pLVAD and external pacing. Amiodarone and lidocaine were used as antiarrhythmic agents. However, no inotropic agents were used in this case. (Fig. 3 & Supplemental Fig. 1) After the introduction of VA-ECMO, pLVAD, and external pacing, the circulatory status stabilized, and blood tests showed an improving trend in CK-MB and troponin I levels, with no recurrence of deterioration. Blood pressure remained stable, allowing for gradual tapering of norepinephrine and dobutamine over time. Following the initiation of temporary pacing, no recurrence of arrhythmias, including complete atrioventricular block or ventricular fibrillation, was observed despite continued use of lidocaine and amiodarone. (Supplemental Fig. 1) Weaning from mechanical circulatory support was performed in a stepwise manner based on hemodynamic stabilization and recovery of end-organ function. VA-ECMO was discontinued on hospital day 6 after confirmation of stable systemic perfusion despite reduced vasoactive support, preserved right ventricular contractility on frequent bedside echocardiography, and adequate pulmonary oxygenation achievable with mechanical ventilation alone. Subsequently, the pLVAD was removed on hospital day 10 following improvement in left ventricular function and sustained hemodynamic stability that could be maintained with low-dose catecholamine support. Improvement in left ventricular function was confirmed by transthoracic echocardiography, demonstrating recovery of the left ventricular ejection fraction from 22 to 68%, as assessed by the biplane Simpson method. Finally, temporary transvenous pacing was withdrawn on hospital day 11 after recovery of atrioventricular conduction without recurrence of bradyarrhythmias or ventricular arrhythmias. After 3 days of high-dose mPSL treatment, the dose was gradually reduced from 50 mg (1 mg/kg) (Fig. 3). During steroid tapering, no recurrence of clinical symptoms, worsening of blood test results, or deterioration of circulatory status on ECG or echocardiography was observed. A maintenance dose of 5 mg for oral steroid was chosen because of concerns regarding immune-related myocarditis relapse. The patient was discharged on Day 69.

**Fig. 4:**
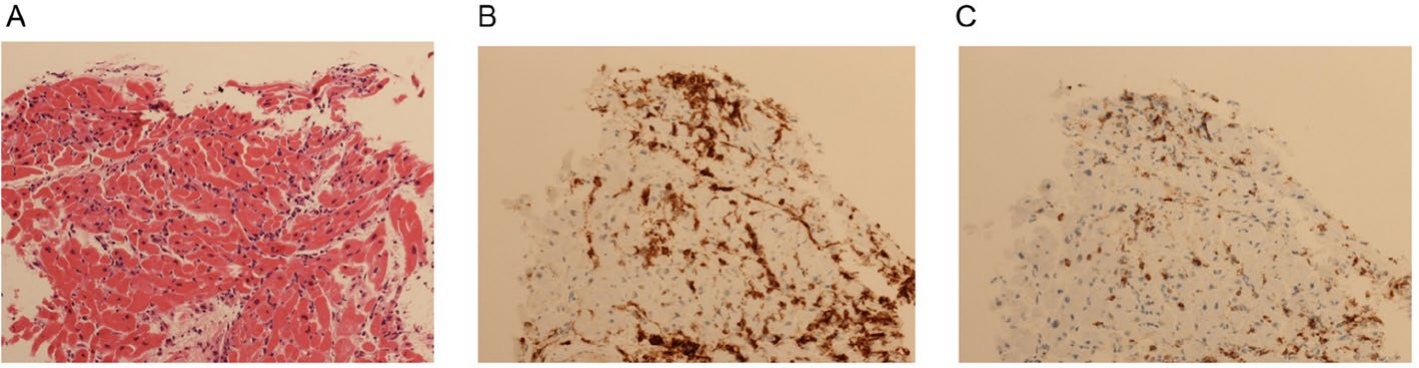
Myocardial biopsy findings. **a** Haematoxylin and eosin (H&E) staining and (**b**, **c**) immunohistochemistry for (**b**) anti-CD4 and (**c**) anti-CD8. H&E-stained myocardial biopsy revealed thin fibers with focal necrosis and moderate lymphocyte infiltration between the myocardial fibers. Immunohistochemistry was used to detect the proportions of CD4 + and CD8 + T cells

Endomyocardial biopsy revealed thin myocardial fibers with focal necrosis and moderate interstitial lymphocytic infiltration. Immunohistochemical staining revealed positivity for CD4, CD8, and CD68, which was consistent with a diagnosis of lymphocytic myocarditis (Fig. 4a–c).

Additionally, considering the possibility of cytokine release syndrome, cytokines IFN-γ, IL-2, IL-6, IL-10, and IL-12 were measured at three time points: before symptom onset, at the emergency department, and after initiation of the steroid treatment. However, only IL-6 levels showed mild fluctuations (Table 1).

**Table 1:** A blood test measuring IL-6 at three time points: before symptom onset, at emergency department, and after the initiation of steroid treatment

	IFN-γ (pg/mL)	IL-2 (pg/mL)	IL-6 (pg/mL)	IL-10 (pg/mL)	IL-12 (pg/mL)
Before symptom onset	< 1.56	< 15.6	83.1	< 0.78	< 0.78
At emergency department	< 1.56	< 15.6	118	1.36	< 0.78
After the initiation of steroid treatment	< 1.56	< 15.6	37.4	2.76	< 0.78

## Discussions and conclusions

We report a case of fulminant immune-related myocarditis in which the patient survived due to prompt diagnosis and timely invasive treatment. irAEs have been associated with a broad spectrum of toxicities, and although immune-related myocarditis has a low incidence, it has a high mortality rate. Several risk factors for immune-related myocarditis have been reported; particularly, ipilimumab plus nivolumab, which was used in this case, has been associated with a significantly higher risk of immune-related myocarditis compared to other ICI therapies [4]. In addition, combination ICI therapy is associated with greater mortality than monotherapy, with ipilimumab plus nivolumab-induced myocarditis reportedly fatal in 66.75% of cases [5]. Immune-related myocarditis is typically arrhythmogenic and not accompanied by left ventricular systolic dysfunction (LVSD) [6]. Current evidence suggest that shared myocardial and tumor antigens trigger T-cell- and macrophage-mediated infiltration of both the myocardium and the cardiac conduction system, producing conduction disturbances that differ fundamentally from the predominantly myocyte-centered inflammation of viral myocarditis [7].

In our patient, several features supported the diagnosis of immune-related myocarditis: (i) recent exposure to ipilimumab plus nivolumab combination therapy, (ii) early onset of events following the initiation of ICI therapy, and (iii) predominant conduction abnormalities without LVSD. Viral myocarditis, which is more common in younger individuals and primarily driven by an inflammatory response associated with infection, was considered less likely.

According to the National Comprehensive Cancer Network guidelines, the initial treatment for immune-related myocarditis of Grade 3 or Grade 4 is ICI discontinuation and administration of high-dose mPSL (1000 mg/day for three days) [8]. Early administration of high-dose corticosteroids leads to a lower incidence of cardiac adverse events, and in particular, therapeutic intervention within 24 h of hospitalization contributes to improved prognosis [9]. Second-line treatment options remain unproven. Currently, the use of intravenous immunoglobulin, mycophenolate, infliximab, tocilizumab, alemtuzumab, and abatacept has been reported [10]. However, infliximab should be avoided in patients with New York Heart failure of NYHA class III/IV congestive heart failure, as it has been reported to significantly increase the risk of cardiovascular mortality. Therefore, their use is considered undesirable [11].

Several hours after initiating methylprednisolone therapy, our patient developed a hemodynamic collapse, necessitating invasive VA-ECMO, pLVAD, and temporary transvenous cardiac pacing. Thereafter, the hemodynamics stabilized, and cardiac biomarkers such as CK-MB and troponin I steadily improved, suggesting that the steroid therapy was effective. Steroids alone exert an anti-inflammatory effect; however, a circulatory support device is required until this effect becomes apparent. We tapered oral corticosteroids very gradually with 5 mg decrements, in view of the reported re-emergence of immune-related myocarditis after rapid withdrawal of corticosteroids [12]. Treatment success was achieved with steroid therapy alone in this case; however, next treatment options were also considered. The patient’s transient elevation of serum inflammatory cytokines (IL-6) raised the possibility of concomitant cytokine release syndrome; hence anti-IL-6/IL-6R therapy, such as tocilizumab, would have been our next treatment option.

In patients with advanced-stage malignancies, these interventions are highly invasive, and the indication for mechanical circulatory support such as VA-ECMO and pLVAD represents an important clinical issue that requires careful consideration of both oncological prognosis and the severity of treatment-related toxicity. In this case, the decision to initiate invasive treatment, including VA-ECMO and pLVAD, was based on three key considerations. First, the patient’s cardiac dysfunction was attributed not to progression of the malignancy but to immune-related myocarditis, a potentially reversible condition. Second, at the time of ECMO initiation, the malignant pleural mesothelioma was well controlled, and the patient’s short-term prognosis was determined primarily by myocarditis rather than by the underlying cancer. Third, multidisciplinary discussions between the cardiology and oncology teams concluded that aggressive circulatory support was clinically appropriate from a lifesaving perspective. Based on these considerations, invasive treatment including VA-ECMO and pLVAD was initiated.

In conclusion, we experienced a case of malignant pleural mesothelioma in which early high-dose steroid therapy—within approximately six hours of symptom onset— combined with aggressive mechanical circulatory support enabled survival from fulminant ipilimumab + nivolumab induced myocarditis in a patient with malignant mesothelioma. Rapid and close multidisciplinary coordination between the oncology and cardiology teams is crucial. IrAEs can be life threatening and may be initially managed by non-oncology specialists. The broad dissemination of such clinical experiences is crucial for improving non-oncology specialists' awareness of irAEs and, consequently, patient outcomes.

## Supplementary Information

Supplementary Material 1: Supplemental Figure1. This graph illustrates the relationships among hemodynamic parameters, the use of vasopressors and antiarrhythmic agents, VA-ECMO, pLVAD, and external pacing.

Supplementary Material 2.

## Data Availability

Data is provided within the supplementary information. The datasets used and analyzed during the current study are available from the corresponding author on reasonable request.

## Abbreviations

ICI: Immune checkpoint inhibitors
irAE: Immune-related adverse event
CK-MB: Creatine kinase-MB
CT: Computed tomography
ECG: Electrocardiography
mPSL: Methylprednisolone
VA-ECMO: Venoarterial extracorporeal membrane oxygenation
pLVAD: Percutaneous left ventricular assist device
LVSD: Left ventricular systolic dysfunction
