# Supplementary figures and images for: Recovery from fulminant immune-related myocarditis induced by ipilimumab plus nivolumab in malignant pleural mesothelioma: a case report
Source: BMC Pulm Med. 2026 Mar 10;26:182. doi: 10.1186/s12890-026-04193-3 (PMC13088471; doi:10.1186/s12890-026-04193-3)

Supplemental  
Figure1

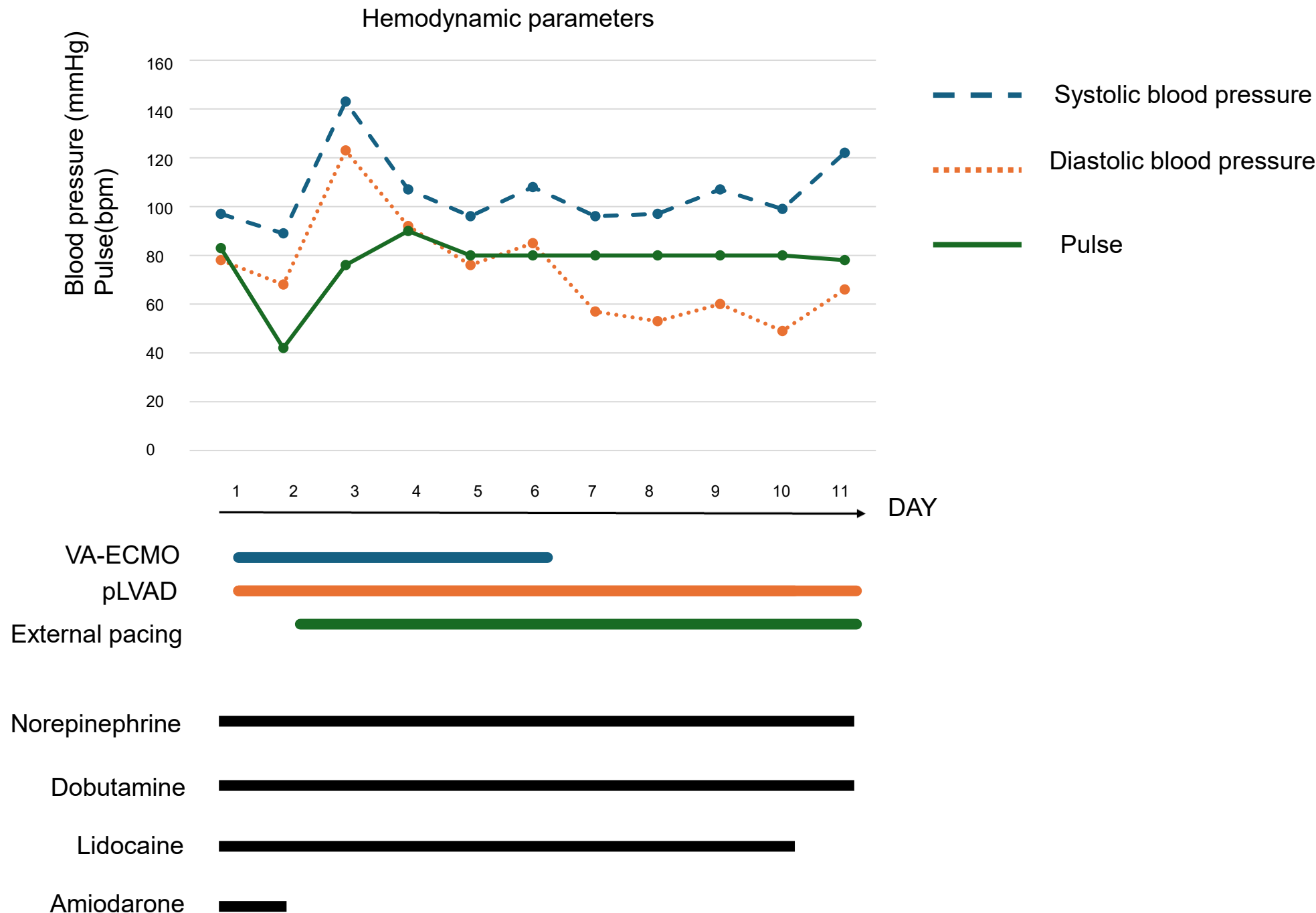

Supplement: Supplementary file 1 — Supplementary Material 1: Supplemental Figure1. This graph illustrates the relationships among hemodynamic parameters, the use of vasopressors and antiarrhythmic agents, VA-ECMO, pLVAD, and external pacing. [file 12890_2026_4193_MOESM1_ESM.pdf]
